# Mapping the cognitive landscape: a pilot study of domain-specific outcomes following frontal lobe resection in children with epilepsy

**DOI:** 10.3389/fneur.2026.1782715

**Published:** 2026-03-19

**Authors:** Feng Shuo, Xu Junhan, Ji Xinna, Chen Qian

**Affiliations:** Capital Center for Children's Health, Capital Medical University, Beijing, China

**Keywords:** antiseizure medications, cognitive outcomes, frontal lobe epilepsy, lateralization, neuropsychological assessment, pediatric neurosurgery

## Abstract

**Background:**

Pediatric frontal lobe epilepsy surgery can achieve seizure freedom, but cognitive outcomes are heterogeneous and poorly predicted by global measures. This pilot study aimed to implement a domain-specific cognitive assessment protocol and perform an exploratory analysis to identify clinical variables that may influence postoperative outcomes, thereby generating hypotheses for future research.

**Methods:**

This retrospective pilot study included 17 pediatric patients with drug-resistant epilepsy (DRE) who underwent frontal lobe resection. All patients completed the Computerized Cognitive Testing in Epilepsy (CCTE) battery before and at least 6 months after surgery, assessing memory, attention, language, mathematics, reasoning, visuospatial skills, and psychomotor speed. Domain-specific change was calculated. An exploratory analysis examined associations with antiseizure medication (ASM) burden, age at onset, age at surgery, epilepsy duration, and resection side.

**Results:**

Implementation of the protocol was feasible (100% completion). Cognitive outcomes were highly heterogeneous and domain-specific. Exploratory analyses suggested trends warranting future investigation: a higher preoperative ASM load was associated with a decline in numerical reasoning, while a lack of postoperative ASM reduction was linked to declines in associative memory and psychomotor speed. Earlier seizure onset and younger surgical age were observed in patients with visuospatial deficits, whereas older surgical age and longer epilepsy duration were observed in those with language impairments. A paradoxical trend indicated that higher baseline performance in several domains was associated with greater postoperative decline. A pronounced lateralization effect was noted, with visuospatial deficits universal after right-sided resections but not after left-sided resections.

**Conclusion:**

This pilot study demonstrates the feasibility and clinical value of domain-specific cognitive assessment, revealing heterogeneous outcomes not captured by global measures. The exploratory findings generate specific hypotheses regarding the roles of ASM management, developmental timing, baseline cognition, and surgical laterality. These results provide a foundation for larger, prospective studies to build predictive models for personalized postoperative rehabilitation.

## 1 Introduction

Epilepsy is a chronic neurological disorder characterized by recurrent, unprovoked seizures resulting from abnormal neuronal activity in the brain. In children, epilepsy is particularly consequential, disrupting critical periods of cognitive development and imposing substantial long-term psychosocial burdens on patients and their families (1, 2). Despite advances in antiseizure medications (ASMs), approximately 20–30% of pediatric patients develop drug-resistant epilepsy (DRE), representing a formidable clinical challenge (3, 4). For DRE localized to the frontal lobe, a region accounting for roughly 25% of pediatric focal epilepsy and implicated in high-order cognitive functions, resective surgery is a well-established therapeutic mainstay (5–7).

However, seizure control alone is an insufficient measure of success in the developing brain. The frontal lobe is a cornerstone of executive function, working memory, and processing speed (8, 9). Resection, while often curative for seizures, carries the inherent risk of disrupting these developing cognitive networks. Current paradigms for evaluating surgical outcomes disproportionately rely on global measures, such as intelligence quotient (IQ), which can mask significant, domain-specific cognitive changes that are more relevant to daily functioning and academic progress (10, 11). A critical gap exists in the routine application of detailed, multi-domain neuropsychological assessments that can capture this heterogeneity and inform personalized rehabilitation.

Significant challenges in studying this population exacerbate this gap. Pediatric frontal lobe epilepsy cohorts are inherently heterogeneous in etiology, encompassing structural abnormalities, genetic mutations, and acquired injuries, and recruiting large, homogeneous samples for definitive studies is difficult (12, 13). Consequently, the literature lacks granular data on how specific cognitive domains are affected and what perioperative factors might modulate these outcomes. Key clinical variables, such as ASM burden, age at intervention, epilepsy duration, and surgical laterality, are frequently discussed but remain poorly quantified in relation to specific functions, including visuospatial reasoning and associative memory (14, 15).

Therefore, well-designed pilot studies are essential for defining methodologies, demonstrating feasibility, and generating focused hypotheses for future large-scale research. By concentrating on a carefully characterized cohort, such studies can provide the preliminary evidence necessary to justify more complex, multi-center investigations (16).

The present study was conceived as a hypothesis-generating pilot with two primary aims: (1) to demonstrate the feasibility and clinical value of a detailed, computerized neuropsychological battery in capturing domain-specific cognitive profiles before and after frontal lobe resection in children; and (2) to perform an exploratory analysis identifying preliminary associations between key clinical variables (ASM load, age parameters, surgical laterality, and baseline cognitive performance) and changes in specific cognitive domains. We hypothesized that this fine-grained approach would reveal heterogeneous cognitive outcomes not reflected in global scores and would yield specific, testable hypotheses to guide future prospective research.

## 2 Methods

### 2.1 Study design and rationale

This was a retrospective, single-center, hypothesis-generating pilot study. Recognizing the challenges in recruiting large, homogeneous cohorts for pediatric frontal lobe epilepsy surgery, we focused on a well-characterized subgroup to maximize internal validity for this initial exploration. To maximize internal validity for this initial exploration, we focused on a well-characterized subgroup: school-aged children (6–18 years) with unilateral frontal lobe DRE who had the cognitive capacity to complete standardized computerized testing. We acknowledge that this selective approach limits the generalizability of our findings, but it provides a necessary foundation for defining methodologies and generating specific research hypotheses.

### 2.2 Patient cohort

**Figure 1:**
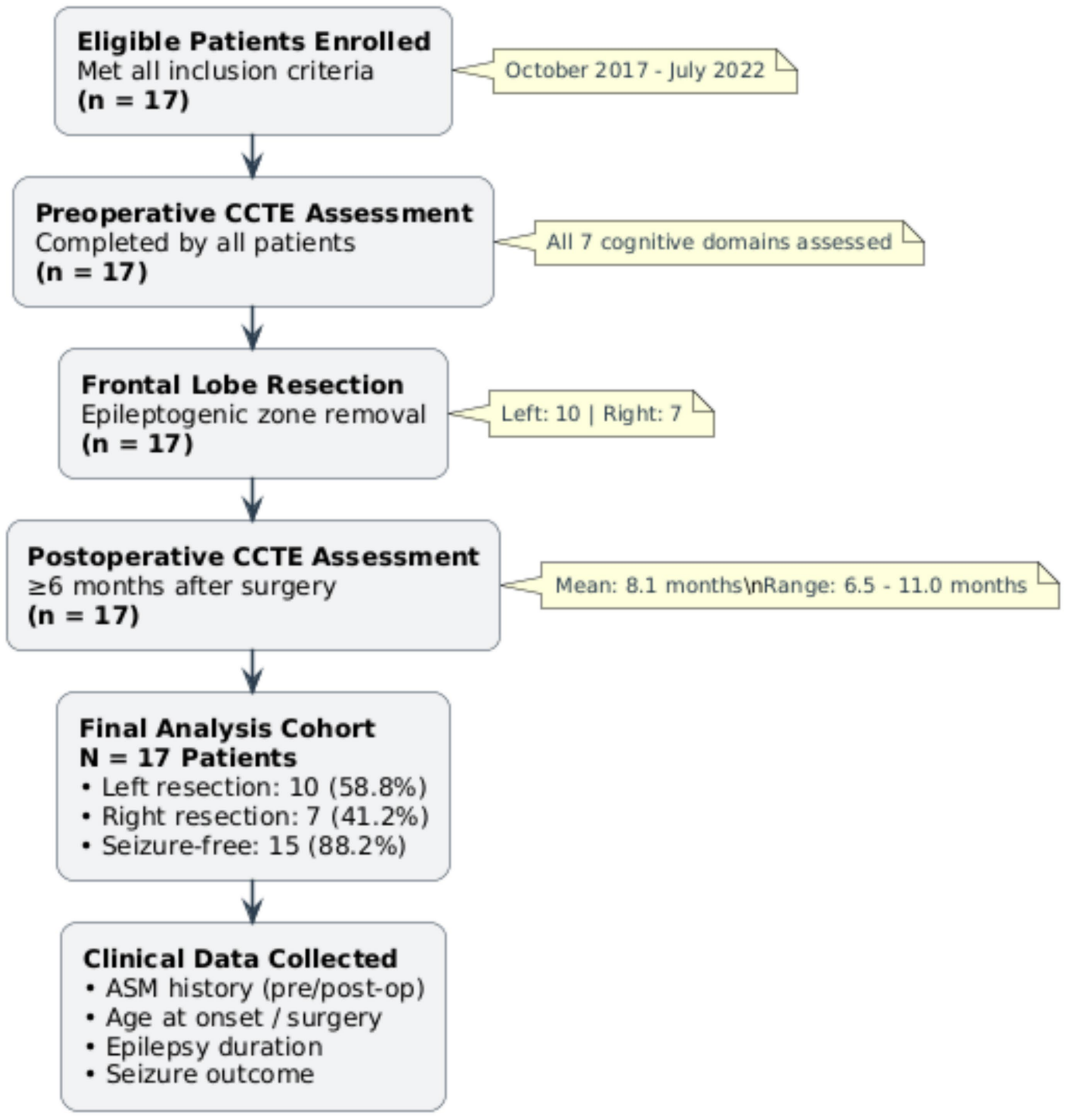
Study flowchart. DRE, drug-resistant epilepsy; EZ, epileptogenic zone; CCTE: computerized cognitive testing in epilepsy; ASM, antiseizure medication.

The study analyzed 17 pediatric patients (12 males, 5 females) with DRE (ILAE criteria) who underwent resection of the frontal lobe epileptogenic zone at the Capital Institute of Pediatrics between October 2017 and July 2022. A multidisciplinary epilepsy surgery team confirmed unilateral frontal lobe localization preoperatively through a standardized protocol including prolonged video-EEG monitoring and magnetic resonance imaging (MRI). The patient enrollment workflow is summarized in Figure 1.

#### 2.2.1 Inclusion criteria

Patients were included if they met all the following: (1) age between 6 and 18 years at surgery; (2) diagnosis of DRE; (3) comprehensive preoperative evaluation confirming a unilateral frontal lobe epileptogenic zone; (4) normal or corrected vision/hearing and sufficient motor function to operate a computer; (5) ability to understand task instructions; and (6) availability of complete preoperative and postoperative cognitive assessments.

#### 2.2.2 Exclusion criteria

Patients were excluded for any of the following: (1) epileptogenic zone extending beyond the frontal lobe or evidence of multifocality; (2) significant physical or cognitive impairment precluding valid computerized test completion; (3) comorbid intellectual disability (IQ < 70) or global developmental delay, as these conditions could confound the assessment of surgery-specific cognitive changes.

### 2.3 Clinical variables

Demographic and clinical data were extracted from medical records. Variables included sex, age at seizure onset, age at surgery, epilepsy duration (calculated as age at surgery minus age at onset), surgical laterality (left vs. right), and ASM history. ASM data included the number of medications at the preoperative baseline and at the postoperative follow-up assessment, as well as a binary indicator of whether the total number of ASMs was reduced postoperatively.

### 2.4 Cognitive assessment

Cognitive function was assessed using the Computerized Cognitive Testing in Epilepsy (CCTE) battery (16). The CCTE evaluates seven cognitive domains through 13 standardized tasks: (1) Memory (verbal working memory, verbal associative learning/recall, mathematical associative learning/recall); (2) Attention (visual tracking); (3) Language (word discrimination, Chinese rhyming); (4) Mathematics (simple arithmetic, numerical magnitude comparison); (5) Reasoning (numerical sequence reasoning, figural analogy reasoning); (6) Visuospatial Processing (3D mental rotation); and (7) Psychomotor Speed (choice reaction time).

Before testing, trained personnel provided standardized instructions and practice trials. Responses were primarily made via the “P” and “Q” keys (visual tracking used a mouse). The system automatically recorded accuracy and reaction time. The full battery required approximately 50 min, with patient-determined breaks. All participants completed testing within one month prior to surgery and at a postoperative follow-up visit (minimum interval: 6 months).

### 2.5 Quantification of cognitive change

For each CCTE task, individual change was calculated as a percentage using the following Equation 1:


$$\text{Percent}\;\text{Change}\;(\%)=\frac{\begin{array}{l} \text{Postoperative Score}\\ -\text{Preoperative Score} \end{array}}{\text{Preoperative Score}}\;\;x\;100\;\;$$ (1)

Based on this metric, postoperative performance on each task was classified as: Improved (≥ + 10% change), Stable (−10 to +10% change), or Declined (≤ − 10% change). For subsequent analyses, a binary outcome was derived where “Declined” was classified as postoperative cognitive impairment for that specific domain, while “Improved” or “Stable” were classified as no impairment.

### 2.6 Statistical analysis

Analyses were conducted using SPSS 27.0 (IBM Corp.). Given the exploratory nature of this pilot study and the limited sample size (*n* = 17), the analysis is explicitly descriptive and intended to identify patterns and generate hypotheses rather than to test definitive associations. Descriptive statistics are presented as mean ± standard deviation (SD) for continuous variables and as counts (percentages) for categorical variables. Group comparisons are presented for transparency but should be interpreted with extreme caution due to the high risk of Type I and Type II errors. No corrections for multiple comparisons were applied, in line with the exploratory aim of the study.

## 3 Results

### 3.1 Feasibility, cohort characteristics, and seizure outcomes

The implementation of a detailed cognitive assessment protocol was feasible in this pediatric cohort undergoing frontal lobe epilepsy surgery. All 17 consecutively eligible patients successfully completed the full CCTE battery both before and after surgery, yielding a 100% assessment completion rate.

The demographic and clinical characteristics of the cohort are detailed in Table 1. Briefly, the cohort had a mean epilepsy duration of 4.3 years prior to surgery. Critically, seizure outcomes were favorable: 15 patients (88.2%) were seizure-free (Engel Class Ia), and the remaining 2 (11.8%) experienced a > 50% reduction in seizure frequency at the time of cognitive follow-up (at a mean follow-up of 8.1 months). The cohort was balanced between left (*n* = 10) and right (*n* = 7) frontal resections. As anticipated in this modest sample, no statistically significant differences in baseline characteristics were found between the left and right resection groups (all *p* > 0.05, Table 1).

**Table 1:** Demographic and clinical characteristics of the pilot cohort (*N* = 17).

Variable	Total cohort (*N* = 17)	Left frontal resection (*n* = 10)	Right frontal resection (*n* = 7)	*p*-value
Sex, male/female	12/5	8/2	4/3	0.593
Age at seizure onset (years)	5.17 ± 2.43	5.94 ± 2.49	4.07 ± 2.01	0.122
Age at surgery (years)	9.42 ± 2.61	9.74 ± 2.59	8.97 ± 2.78	0.568
Epilepsy duration (years)	4.25 ± 2.25	3.80 ± 1.57	4.90 ± 2.30	0.399
Preoperative ASM number	1.59 ± 1.09	1.60 ± 1.09	1.57 ± 1.13	0.959
Postoperative ASM number	2.00 ± 0.87	2.10 ± 0.99	1.86 ± 0.69	0.561
ASM reduction post-op, yes/no	5/12	2/8	3/4	0.593
Post-op follow-up (months)	8.12 ± 2.39 (6.5–11.0)	8.05 ± 2.56	8.21 ± 2.27	0.893

ASM, anti-seizure medication. Data are mean ± SD or count. Range is shown in parentheses for the total cohort follow-up. *p*-values from independent *t*-tests (continuous) or Fisher’s exact test (categorical) are provided for descriptive comparison between resection groups and should be interpreted with caution due to the small sample size.

### 3.2 Heterogeneity of domain-specific cognitive outcomes

Consistent with our hypothesis, postoperative cognitive changes were highly heterogeneous and domain-specific. No uniform pattern of decline emerged across the cohort. To illustrate this individual variability, Table 2 presents the percent change in four key cognitive domains for each patient, ordered by surgical side. Patients commonly showed a decline in one domain alongside stability or improvement in another (e.g., Patient L-01 declined in visuospatial reasoning but improved in verbal memory). This domain-specific heterogeneity highlights the limitations of global cognitive scores and underscores the clinical value of detailed, multi-domain profiling.

**Table 2:** Individual patient profiles: percent change in selected cognitive domains.

Patient ID	Resection side	Verbal associative memory (%)	Word discrimination (%)	3D Mental rotation (visuospatial) (%)	Choice reaction time (%)
L-01	Left	**+18**	-7	-5	+4
L-02	Left	**−42**	+2	+8	**−19**
L-03	Left	−5	**−32**	−3	+1
L-04	Left	**+22**	0	**−15**	+6
L-05	Left	**−38**	+5	+12	−8
L-06	Left	+3	−4	**−28**	**+15**
L-07	Left	**+11**	**−25**	0	−2
L-08	Left	−8	+9	**−12**	**−21**
L-09	Left	**−55**	+1	+5	+3
L-10	Left	+2	**−18**	−8	**+11**
R-01	Right	−3	+6	**−48**	**−12**
R-02	Right	**+25**	−2	**−35**	+2
R-03	Right	**−15**	+4	**−40**	**−25**
R-04	Right	+7	**−22**	**−52**	+7
R-05	Right	0	+3	**−38**	**−16**
R-06	Right	**+30**	−1	**−45**	+5
R-07	Right	+4	+8	**−50**	**−22**

Data show percent change from pre- to postoperative assessment (Positive, improvement; Negative, decline). Bold values highlight changes ≥│10%│. Patient IDs are anonymized (L, left resection; R, right resection). This table visually demonstrates the heterogeneous, domain-specific nature of cognitive outcomes.

### 3.3 Exploratory analysis of clinical associations

Given the observed heterogeneity, we performed an exploratory analysis to identify patterns that may warrant investigation in larger studies. Due to the small sample size and the multiple comparisons inherent in domain-specific analysis, the following observations are presented as preliminary descriptive patterns intended for hypothesis generation only. They should not be interpreted as clinically informative relationships.

#### 3.3.1 Anti-seizure medication (ASM) patterns

Preliminary patterns were noted linking ASM burden to outcomes in specific domains (Table 3). Notably, a postoperative decline in mathematical associative learning and psychomotor speed (reaction time) appeared to be associated with a lack of postoperative ASM reduction. Furthermore, a higher preoperative ASM load was observed in patients who later declined in numerical reasoning.

**Table 3:** Exploratory analysis of ASM variables and domain-specific cognitive outcome.

Cognitive task (domain)	ASM variable	Impaired group (n)	Non-impaired group (*n*)
Mathematical associative learning (memory)	Post-op ASM number	2.57 ± 0.79 (*n* = 8)	1.63 ± 0.74 (*n* = 9)
Mathematical associative learning (memory)	ASM reduction (Yes/No)	0/8 (*n* = 8)	5/4 (*n* = 9)
Numerical reasoning (reasoning)	Pre-op ASM number	2.43 ± 0.53 (*n* = 7)	1.00 ± 0.94 (*n* = 10)
Choice reaction time (psychomotor speed)	ASM reduction (Yes/No)	0/8 (*n* = 8)	5/4 (*n* = 9)

Data are mean ± SD or count. These analyses are exploratory and descriptive, intended for hypothesis generation only.

#### 3.3.2 Age-related variables

Distinct patterns emerged concerning age and epilepsy history in this small cohort (Table 4). On average, a decline in visuospatial function (3D mental rotation) was observed in patients with earlier seizure onset and younger surgical age. Conversely, a decline in language function (word discrimination) was observed, on average, in patients who were older at surgery and had a longer history of epilepsy.

**Table 4:** Exploratory analysis of age and epilepsy duration variables.

Cognitive task (domain)	Clinical variable	Impaired group (*n*)	Non-impaired group (*n*)
3D Mental rotation (visuospatial)	Age at onset (years)	3.92 ± 1.68 (*n* = 10)	6.58 ± 2.45 (*n* = 7)
3D Mental rotation (visuospatial)	Age at surgery (years)	8.16 ± 1.65 (*n* = 10)	10.85 ± 2.85 (*n* = 7)
Word discrimination (language)	Age at surgery (years)	10.61 ± 2.66 (*n* = 7)	7.73 ± 1.40 (*n* = 10)
Word discrimination (language)	Epilepsy duration (years)	5.17 ± 2.24 (*n* = 7)	2.94 ± 1.60 (*n* = 10)

Data are mean ± SD. These analyses are exploratory and descriptive, intended for hypothesis generation only.

#### 3.3.3 Baseline cognitive performance

An unexpected pattern emerged in this pilot cohort, suggesting that higher preoperative performance in certain domains appeared more frequently in patients who experienced postoperative decline (Table 5). This preliminary observation requires validation in larger studies. The mean preoperative scores of the impaired groups were notably higher than those of the non-impaired groups in verbal associative memory, word discrimination, Chinese rhyme judgment, and choice reaction time.

**Table 5:** Preoperative cognitive scores by postoperative outcome group.

Cognitive task (domain)	Impaired group (*n*)	Non-impaired group (*n*)
Verbal associative learning (memory)	71.86 ± 18.65 (*n* = 7)	22.00 ± 14.14 (*n* = 10)
Word discrimination (language)	42.90 ± 23.00 (*n* = 7)	18.00 ± 17.80 (*n* = 10)
Chinese rhyme (language)	41.00 ± 27.87 (*n* = 7)	17.38 ± 12.75 (*n* = 8)
Choice reaction time (psychomotor Speed)	53.13 ± 17.18 (*n* = 8)	27.44 ± 18.53 (*n* = 9)

Data are mean ± SD of raw preoperative task scores. Group sizes (n) are small and vary due to missing data in one task. These analyses are exploratory and descriptive, intended for hypothesis generation only.

#### 3.3.4 Surgical laterality and visuospatial function

A notable and consistent pattern was observed concerning the surgical side. While a decline in visuospatial function (3D mental rotation) occurred in a subset of patients with left-sided resections (3 of 10, 30%), it was observed in all patients (7 of 7, 100%) who underwent right frontal resection (*p* = 0.010, Fisher’s exact test). This stark contrast suggests a potentially strong lateralized effect that merits focused examination in future studies. These preliminary observations require validation in larger, prospective cohorts before any clinical recommendations can be considered.

## 4 Discussion

This pilot study provides preliminary, hypothesis-generating observations that require confirmation in larger cohorts. The interpretations offered below should be read with this foundational caveat in mind. This pilot study demonstrates that implementing a detailed, domain-specific cognitive assessment protocol in children undergoing frontal lobe epilepsy surgery is not only feasible but also reveals critical heterogeneity in outcomes that global measures would obscure. Consistent with our hypothesis, we observed no uniform cognitive trajectory; instead, each patient exhibited a unique profile of improvements and declines across distinct cognitive domains (Table 2). This core finding highlights a significant clinical reality: seizure freedom does not guarantee uniform cognitive preservation, and rehabilitation strategies must be tailored to individual needs (11). The exploratory analyses in our modest cohort further generated specific, testable hypotheses regarding the roles of ASM management, age-related factors, baseline cognition, and surgical laterality, each of which warrants rigorous investigation in larger, prospective studies.

Our exploratory data suggest trends where a higher postoperative ASM burden and lack of ASM reduction were associated with a greater decline in specific domains, such as mathematical learning and psychomotor speed. This observation aligns with a growing body of literature indicating that ASMs, while essential for seizure control, can exert negative cognitive effects, and that judicious postoperative reduction may confer cognitive benefit (17–20). The optimal timing of ASM withdrawal after successful epilepsy surgery remains an important clinical question. Studies such as the TimeToStop collaborative initiative have demonstrated that early ASM discontinuation is feasible in many children after epilepsy surgery and may be associated with improved developmental outcomes (21). Our findings extend this concept to the pediatric frontal lobe population, highlighting ASM load as a potentially modifiable factor. However, these observations should be interpreted with caution, as ASM burden may also serve as a proxy for underlying disease severity, which we could not fully account for in this pilot cohort. Future research must move beyond medication counts to analyze the specific cognitive profiles of individual ASMs, their doses, and the optimal timing for reduction, with prospective designs like those pioneered by the TimeToStop studies.

The complex relationship between developmental timing and cognitive outcome was evident in our exploratory trends. Earlier seizure onset and surgery were tentatively linked to visuospatial deficits, possibly reflecting disruption during a sensitive period for the maturation of fronto-parietal networks (14, 22). While this interpretation is biologically plausible, it remains speculative in the absence of direct mechanistic or longitudinal evidence. These patterns may also be confounded by etiology-specific developmental trajectories, which were not available for analysis in this pilot study. Conversely, older age at surgery and longer epilepsy duration were associated with language processing declines. This pattern may reflect cumulative effects of chronic epilepsy, along with prolonged ASM exposure, which can impair cognitive systems essential for language, such as attention and processing speed, and may exploit diminished neuroplasticity with delayed intervention (23, 24). However, this interpretation, while consistent with existing literature, requires confirmation in longitudinal studies with comprehensive developmental assessments. These opposing patterns underscore that there is no single ‘optimal’ age for surgery from a cognitive standpoint; risks are domain-specific and must be balanced against the escalating cognitive toll of uncontrolled seizures.

A particularly intriguing finding was the pattern wherein higher preoperative performance in verbal memory, language, and processing speed appeared more frequently in patients who experienced postoperative decline in those same domains. This paradoxical pattern has been reported in adult epilepsy surgery cohorts and can be interpreted through the lens of neural reorganization (8). The ‘functional adequacy’ model posits that well-developed, high-performing cognitive networks may be more functionally specialized and reliant on the resected tissue, thus rendering them more vulnerable to disruption. In contrast, the ‘cognitive reserve’ model would predict better recovery. We present these neurobiological models as potential explanations for our observations; however, they remain speculative without direct evidence of network reorganization, such as pre- and postoperative functional imaging. Furthermore, baseline cognitive differences likely vary across etiological subgroups, a factor we could not examine in this pilot study. This highlights the inadequacy of assuming that a strong preoperative cognitive profile guarantees a favorable outcome, though this interpretation requires confirmation in larger, well-characterized cohorts.

The most consistent finding in our cohort was the profound vulnerability of visuospatial function, measured by 3D mental rotation, following right frontal resection. While left-sided resections led to a decline in a subset of patients, impairment was universal after right-sided surgery. This strongly aligns with the established right-hemisphere specialization for visuospatial processing and mental rotation (25). It suggests that the right frontal lobe plays a critical and non-redundant role in coordinating these functions, making them highly susceptible to disruption from right-sided surgery. Nevertheless, even this observation should be interpreted with caution given the small number of patients in the right-sided group (*n* = 7). This clear lateralization effect provides a robust hypothesis for future studies and has immediate implications for preoperative counseling and postoperative rehabilitation planning. We emphasize that all interpretations offered in this Discussion are preliminary and hypothesis-generating. They are intended to provide a conceptual framework for future research, not to draw definitive conclusions about mechanisms or clinical practice.

### 4.1 Limitations and future directions

This study has several important limitations that precisely define the necessary next steps for this field of inquiry. First and foremost, the small sample size (*n* = 17) is the principal constraint, limiting statistical power and precluding multivariate analyses to disentangle confounding variables. The findings are explicitly presented as hypothesis-generating trends, not definitive associations. Second, the retrospective design and mean follow-up of 8 months are insufficient to capture long-term cognitive trajectories, which may evolve during adolescence (26). Third, by design, our cohort included only children able to complete computerized testing, excluding those with significant intellectual disability; thus, the findings may not generalize to the entire pediatric surgical population. Fourth, as highlighted by the reviewer, we were unable to provide a descriptive summary of underlying etiologies (e.g., focal cortical dysplasia, tumor, genetic causes) or specific antiseizure medication regimens. Due to the retrospective nature of this pilot study, these data were not consistently available in a quantifiable format for all patients. This represents a significant limitation, as etiological heterogeneity may confound the interpretation of our exploratory findings. For example, observed associations with ASM burden could reflect disease severity rather than medication effects, and age-related patterns may be driven by etiology-specific developmental trajectories. Future prospective studies must prioritize systematic collection of etiological data and detailed medication histories to enable stratified analyses and reduce confounding.

Most critically, as reviewers highlighted, this pilot analysis was necessarily limited to readily accessible clinical variables. Formal assessment of language dominance (e.g., via fMRI or Wada testing) was not available, which may affect the interpretation of lateralization effects on language outcomes. In addition, detailed electroclinical data, including specific seizure types (focal, focal-to-bilateral tonic–clonic, or generalized), the occurrence of status epilepticus, and comprehensive EEG characteristics (topography, rhythms, sleep organization), were not systematically collected in this retrospective cohort. To build upon the hypotheses generated here, future prospective, multi-center studies must systematically incorporate more granular predictors. These include: (1) Etiology and histopathology (e.g., focal cortical dysplasia vs. tumor) (12); (2) Quantitative surgical metrics (e.g., resection volume, connectivity of resected tissue via pre-surgical DTI); (3) Electrophysiological data (e.g., extent of the irritative zone, seizure semiology); and (4) Comprehensive electroclinical features, including seizure types and EEG characteristics. Addressing these limitations through collaborative research is crucial for developing accurate predictive models that can inform individualized surgical decision-making and postoperative care (15).

## 5 Conclusion

This pilot study achieved its primary aims: it demonstrated the feasibility and clinical value of detailed domain-specific cognitive assessment after pediatric frontal lobe epilepsy surgery, and it generated focused hypotheses for future research. We showed that cognitive outcomes are heterogeneous and that factors such as ASM burden, age at intervention, epilepsy duration, baseline cognitive performance, and surgical laterality may modulate domain-specific risks. The universal visuospatial decline after right frontal resection is a particularly strong signal warranting confirmation. These preliminary insights underscore the need to move beyond global IQ scores in outcome assessment. They provide a clear methodological and conceptual foundation for designing large-scale, longitudinal studies that can identify modifiable predictors and ultimately pave the way for personalized cognitive prognostication and rehabilitation in this vulnerable population.

## Data Availability

The original contributions presented in the study are included in the article/supplementary material, further inquiries can be directed to the corresponding author.
